# Transcatheter Aortic Valve Replacement Within a Structured Secondary Prevention Framework in Cardiovascular Disease

**DOI:** 10.7759/cureus.105072

**Published:** 2026-03-11

**Authors:** María Jennifer Valle Mena, Maynor Jose Lopez Mendoza, Maria Antonieta Salazar Estrada, Nicolle Contreras Figueroa, Asdrubal Ulloa, Jeilyn Jiron Vindas

**Affiliations:** 1 General Medicine, Área de Salud Upala, Alajuela, CRI; 2 Anesthesiology and Perioperative Medicine, Hospital de las Mujeres Dr. Adolfo Carit Eva (CARITEVA), San José, CRI; 3 Emergency Medicine, Hospital Los Chiles, Los Chiles, CRI; 4 General Medicine, Caja Costarricense de Seguro Social (CCSS), San José, CRI; 5 Gynecologic Oncology, Hospital de las Mujeres Dr. Adolfo Carit Eva (CARIT/EVA), San José, CRI; 6 Obstetrics and Gynecology, Hospital Metropolitano de Atención Especializada (HOMACE), San José, CRI

**Keywords:** aortic stenosis, cardiovascular disease, multimodality imaging, secondary prevention, transcatheter aortic valve replacement, valve durability

## Abstract

Degenerative aortic stenosis represents a frequent and clinically significant manifestation of systemic cardiovascular disease, sharing common risk factors and pathophysiological mechanisms with atherosclerosis, including chronic inflammation, endothelial dysfunction, and calcific remodeling. Its frequent coexistence with comorbid conditions such as coronary artery disease and diabetes mellitus contributes to increased morbidity, mortality, and therapeutic complexity. In recent years, transcatheter aortic valve replacement has emerged as a cornerstone therapy for patients with severe symptomatic aortic stenosis across a wide spectrum of surgical risk, offering substantial improvements in hemodynamic performance, functional status, and quality of life.

This review examines transcatheter aortic valve replacement within the context of secondary cardiovascular prevention. While the procedure effectively corrects valvular obstruction, it does not directly modify the systemic atherosclerotic and metabolic processes that drive long-term cardiovascular risk. Emphasis is placed on comprehensive preprocedural evaluation, including multimodality imaging with transthoracic echocardiography, cardiac magnetic resonance, computed tomography, and transesophageal echocardiography, which together support accurate phenotyping, risk stratification, and procedural planning as determinants of downstream outcomes.

Beyond procedural success, transcatheter aortic valve replacement is associated with favorable effects on cardiovascular prognosis, including reductions in heart failure-related hospitalizations. However, as survival improves, residual cardiovascular risk increasingly reflects the impact of systemic comorbidities and myocardial vulnerability rather than valve-related pathology alone. This shift underscores the need for structured secondary prevention strategies following the intervention.

Optimal long-term management therefore requires aggressive control of cardiovascular risk factors, guideline-directed medical therapy, individualized antithrombotic strategies, and structured follow-up integrating prosthetic valve surveillance with ongoing cardiovascular risk reduction. Transcatheter aortic valve replacement should be viewed not as a secondary prevention therapy in isolation, but as a pivotal structural intervention embedded within a broader, longitudinal cardiovascular prevention framework.

## Introduction and background

Cardiovascular disease remains the leading cause of death worldwide, with aortic stenosis representing a major contributor to morbidity and mortality, particularly within the elderly population [1]. The prevalence of aortic stenosis increases progressively with age, and its clinical relevance is amplified by its frequent coexistence with other cardiovascular conditions. This clustering of comorbidities underscores the need for comprehensive management strategies that address not only valvular disease but also the broader cardiovascular risk profile of affected patients [2].

Degenerative aortic stenosis constitutes an advanced manifestation of systemic cardiovascular disease and is characterized by progressive narrowing of the aortic valve, resulting in increased cardiac workload and, ultimately, heart failure [1]. In its most common form, degenerative tricuspid aortic stenosis, the disease shares mechanistic features with atherosclerosis, including chronic inflammation, endothelial dysfunction, and fibrocalcific remodeling. This pathophysiological overlap reinforces the close association between aortic stenosis and systemic cardiovascular comorbidities such as coronary artery disease and diabetes mellitus [3]. Although bicuspid aortic valve disease represents a distinct anatomical and biological entity, the long-term prognosis of patients with severe stenosis in either morphology is strongly influenced by the presence of systemic cardiovascular disease.

Within this evolving clinical landscape, transcatheter aortic valve replacement has undergone substantial development since its introduction, emerging as a preferred therapeutic option for patients with severe symptomatic aortic stenosis across a broad surgical risk spectrum [4,5]. Continued advances in device design and procedural techniques have significantly enhanced the safety and efficacy of transcatheter aortic valve replacement, expanding its applicability to lower-risk populations and supporting its role within lifetime valve management strategies [5-7].

Beyond relieving valvular obstruction, transcatheter aortic valve replacement profoundly modifies the prognostic trajectory of patients with severe aortic stenosis. By correcting the dominant hemodynamic burden, the procedure shifts long-term outcomes away from valve-related mortality toward events driven by systemic cardiovascular disease, including myocardial dysfunction, coronary artery disease, arrhythmias, and vascular pathology. In this context, secondary cardiovascular prevention does not consist of the valve intervention itself, but becomes increasingly relevant after successful replacement, as residual risk is primarily determined by underlying systemic disease rather than persistent valvular pathology [3,8-10].

The aim of this article is therefore to examine transcatheter aortic valve replacement within a structured secondary cardiovascular prevention framework. Specifically, we analyze how correction of valvular obstruction redefines long-term risk determinants, and why comprehensive management of cardiovascular comorbidities remains essential to optimize both short- and long-term outcomes in contemporary clinical practice.

## Review

Methods

This manuscript was developed as a structured narrative review aimed at synthesizing contemporary evidence on the role of transcatheter aortic valve replacement within a secondary cardiovascular prevention framework in patients with severe aortic stenosis. The methodological approach emphasized conceptual integration, clinical applicability, and interpretation of heterogeneous evidence domains, including imaging strategies, procedural outcomes, prognostic data, and long-term cardiovascular care, rather than quantitative evidence aggregation or adherence to formal systematic review protocols.

A focused literature search was conducted between January 2020 and December 2025 using established scientific databases, including PubMed, ScienceDirect, and the Cochrane Library. High-impact cardiovascular journals were also reviewed to identify influential clinical trials, registry analyses, and consensus-oriented publications relevant to the topic. The search strategy combined controlled vocabulary and free-text terms using Boolean operators to ensure comprehensive coverage. Representative search formulations included combinations such as (“aortic stenosis” OR “degenerative aortic stenosis”) AND (“transcatheter aortic valve replacement” OR “TAVR” OR “transcatheter aortic valve implantation” OR “TAVI”) AND (“secondary prevention” OR “cardiovascular prevention” OR “residual cardiovascular risk”). Additional searches incorporated terms related to multimodality imaging, long-term outcomes, heart failure hospitalization, and valve durability to capture literature spanning diagnostic evaluation, procedural impact, and longitudinal prognosis.

The initial search identified approximately 126 potentially relevant records. After title and abstract screening for relevance and duplication, 68 publications underwent full-text review. Following qualitative appraisal and application of predefined eligibility criteria, 43 peer-reviewed studies were selected for inclusion in the final synthesis.

To enhance transparency and consistency in study selection, predefined eligibility criteria were established a priori to guide identification of clinically relevant studies. These criteria were intended to prioritize high-quality clinical evidence, including randomized controlled trials, large prospective and retrospective cohort studies, registry-based analyses, and systematic reviews, directly aligned with the conceptual scope of this narrative synthesis. The eligibility framework is summarized in Table 1.

**Table 1:** Eligibility criteria for literature inclusion.

Inclusion criteria	Exclusion criteria
Clinical studies evaluating transcatheter aortic valve replacement in patients with severe aortic stenosis	Preclinical studies, including animal or in vitro research, except when cited exclusively for pathophysiological context
Randomized controlled trials assessing clinical outcomes after transcatheter aortic valve replacement	Case reports, narrative opinions, or editorials lacking original quantitative data
Prospective and retrospective cohort studies analyzing mortality, cardiovascular events, functional status, or quality of life	Studies without clearly defined or measurable clinical outcomes
Large registry-based studies reporting short- and long-term outcomes of transcatheter aortic valve replacement	Studies focusing exclusively on surgical aortic valve replacement without comparative or contextual relevance to transcatheter approaches
Systematic reviews and meta-analyses addressing clinical effectiveness, safety, and durability of transcatheter aortic valve replacement	
Studies examining the interaction between transcatheter aortic valve replacement and secondary prevention strategies, including coronary artery disease management and cardiovascular risk factor control	
Publications written in English or Spanish	

No quantitative pooling, meta-analysis, formal risk-of-bias grading, or statistical modeling was performed. Evidence appraisal was interpretative and qualitative in nature, consistent with the narrative design of the review. The authors used OpenAI-based artificial intelligence tools to assist with structural organization and linguistic refinement of the manuscript. All study selection, interpretation, critical appraisal, and final scientific judgments were performed exclusively by the authors.

Aortic stenosis and cardiovascular disease: shared pathophysiological bases

Degenerative aortic stenosis and atherosclerotic cardiovascular disease share common pathophysiological foundations that explain their frequent coexistence and parallel progression. Both conditions are associated with a similar profile of cardiovascular risk factors, including advanced age, hypertension, hyperlipidemia, and diabetes mellitus. These factors contribute to disease development and progression through overlapping mechanisms such as chronic inflammation, endothelial dysfunction, and oxidative stress, which promote structural and functional deterioration of both the arterial wall and the aortic valve [11].

At the molecular level, degenerative tricuspid aortic stenosis and atherosclerosis involve comparable signaling pathways that drive calcific and fibrotic remodeling. Activation of the renin-angiotensin system, dysregulation of Notch signaling, and the involvement of osteogenic mediators contribute to progressive valvular calcification and structural valve degeneration. These molecular mechanisms mirror those observed in atherosclerotic plaque formation and progression, further reinforcing the biological link between the two conditions [11]. Although bicuspid aortic valve disease follows a partially distinct anatomical and developmental trajectory, the long-term clinical impact of severe stenosis in either morphology is strongly influenced by the burden of systemic cardiovascular disease. Clinically, degenerative aortic stenosis frequently coexists with other cardiovascular diseases, most notably coronary artery disease. Up to half of patients with aortic stenosis present with concomitant coronary artery disease, a combination that complicates both diagnosis and management, as overlapping symptoms such as angina and dyspnea may obscure the relative contribution of each condition [12]. In addition, metabolic comorbidities play a critical role in disease trajectory. Type 2 diabetes mellitus has been shown to accelerate the progression of aortic stenosis and is associated with higher rates of adverse outcomes, including heart failure and mortality, compared with patients affected by aortic stenosis alone [13].

The relationship between systemic atherosclerotic burden and aortic valve disease progression further supports this integrated pathophysiological framework. Aortic valve sclerosis and non-severe aortic stenosis have been identified as markers of increased cardiovascular risk, suggesting that the extent of systemic atherosclerosis influences valvular disease evolution [14]. This association is also reflected at the molecular level, as transcriptomic analyses have demonstrated upregulation of atherosclerosis-related pathways within stenotic aortic valves in patients with severe coronary artery disease, indicating that mechanisms traditionally linked to vascular atherosclerosis actively contribute to valvular degeneration [15].

From a prognostic perspective, the coexistence of aortic stenosis with coronary artery disease, diabetes, and other cardiovascular comorbidities has important implications for clinical outcomes and management strategies. Therapeutic decisions, including the choice between surgical and transcatheter aortic valve replacement, are influenced by the severity of coronary artery disease and the patient’s overall clinical profile, reflecting the need for individualized and integrated treatment approaches [16,17]. Importantly, because degenerative aortic stenosis frequently represents a manifestation of systemic cardiovascular disease rather than an isolated valvular disorder, correction of valvular obstruction alone does not eliminate the underlying drivers of long-term risk. This pathophysiological overlap provides the biological rationale for structured secondary cardiovascular prevention following successful valve intervention. Moreover, recognition of these shared mechanisms has opened the possibility of therapeutic repositioning, whereby treatments effective in atherosclerotic disease, such as PCSK9 inhibitors and anti-inflammatory agents, may be explored as potential strategies to target common pathways and potentially influence disease trajectory [11].

Pre-procedure evaluation and patient selection

A comprehensive clinical evaluation is fundamental for accurately determining the severity of aortic stenosis and identifying comorbid conditions that may influence therapeutic decision-making and clinical outcomes. This assessment encompasses a detailed evaluation of symptoms, physical examination findings, and the patient’s medical history, allowing for an integrated understanding of disease burden and functional impact [18].

Within this framework, global cardiovascular risk stratification plays a central role. Assessment of overall cardiovascular risk includes careful evaluation for the presence of coronary artery disease, which represents one of the most frequent comorbidities in patients with aortic stenosis. Approximately half of patients undergoing transcatheter aortic valve implantation present with concomitant coronary artery disease, a factor that can significantly influence procedural planning and post-intervention outcomes [3].

Imaging techniques are integral to this comprehensive assessment. Transthoracic echocardiography serves as the first-line imaging modality for evaluating the severity of aortic stenosis, providing essential anatomical and functional information regarding valve morphology, transvalvular gradients, and ventricular response. Complementary to echocardiography, computed tomography plays a crucial role in detailed anatomical assessment of the aortic valve complex and vascular access routes, and it also contributes to the evaluation of coronary anatomy, in some cases serving as an alternative to conventional coronary angiography [19]. Although invasive coronary angiography remains a standard diagnostic tool, computed tomography coronary angiography is increasingly incorporated into pre-transcatheter aortic valve implantation assessment strategies for coronary evaluation [3].

**Figure 1:**
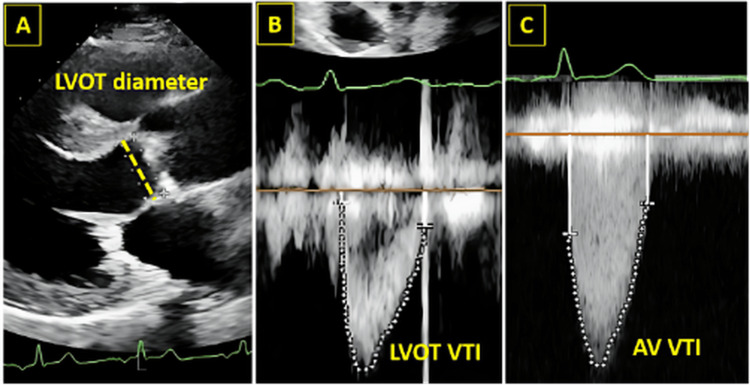
Transthoracic echocardiography illustrating aortic valve area assessment using the continuity equation. Ventricular outflow tract velocity–time integral obtained by pulsed-wave Doppler. (C) Aortic valve velocity–time integral measured by continuous-wave Doppler from the apical five-chamber view.  Figure reproduced from the study by Rezaeian et al. licensed under Creative Commons Attribution-NoDerivatives 4.0 International License [20]. LVOT: Left ventricular outflow tract; VTI: velocity time integral

Figure 1 illustrates the echocardiographic parameters that are fundamental for the hemodynamic assessment of aortic stenosis severity. Using transthoracic echocardiography, the left ventricular outflow tract diameter is measured to calculate stroke volume, while pulsed-wave Doppler is used to obtain the left ventricular outflow tract velocity-time integral and continuous-wave Doppler to derive the aortic valve velocity-time integral. Integration of these measurements allows calculation of the aortic valve area through the continuity equation, providing an objective and reproducible framework for grading stenosis severity and supporting clinical decision-making within the comprehensive diagnostic evaluation.

**Figure 2:**
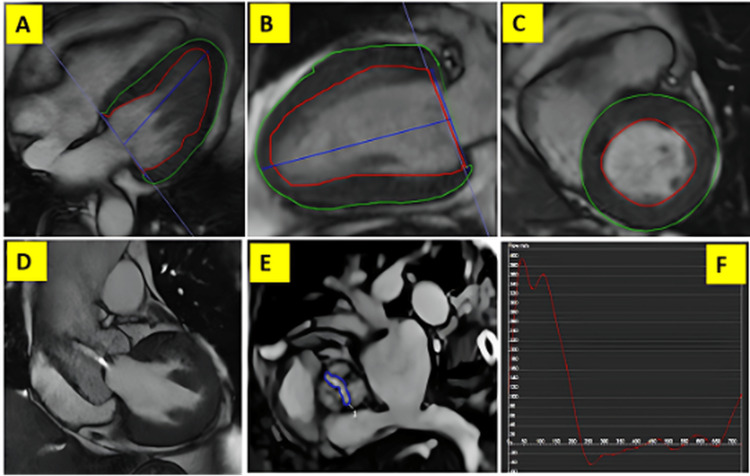
Cardiac magnetic resonance assessment of myocardial deformation and aortic valve area in severe aortic stenosis. Figure reproduced from the study by Rezaeian et al. licensed under Creative Commons Attribution-No Derivatives 4.0 International License [20].

Cardiac magnetic resonance (Figure 2) offers additional insight into myocardial remodeling and aortic valve severity through strain analysis, valve planimetry, and flow assessment, supporting advanced preprocedural evaluation in severe aortic stenosis.

Identification of comorbidities, particularly coronary artery disease, is therefore essential, as these conditions directly influence the selection between surgical and transcatheter treatment approaches. The presence of complex coronary artery disease may require specific revascularization strategies either before or in conjunction with transcatheter aortic valve replacement, further highlighting the importance of an integrated and individualized assessment strategy [16].

Percutaneous aortic valve replacement, procedure and immediate results

Transcatheter aortic valve replacement is performed through the introduction of a catheter via a vascular access site, most commonly using the transfemoral approach, to deliver and implant a prosthetic valve within the diseased native aortic valve. The minimally invasive nature of the procedure has allowed it to be increasingly performed under local anesthesia, a strategy that has been associated with shorter hospital stays and faster recovery times, thereby improving overall patient tolerance and procedural efficiency [21,22]. In parallel, technological advances have contributed substantially to procedural simplification. The introduction of premounted valve systems has eliminated the need for intra-procedural valve crimping, reducing procedural time and minimizing the risk of technical errors, which has further enhanced procedural safety and reproducibility [23].

**Figure 3:**
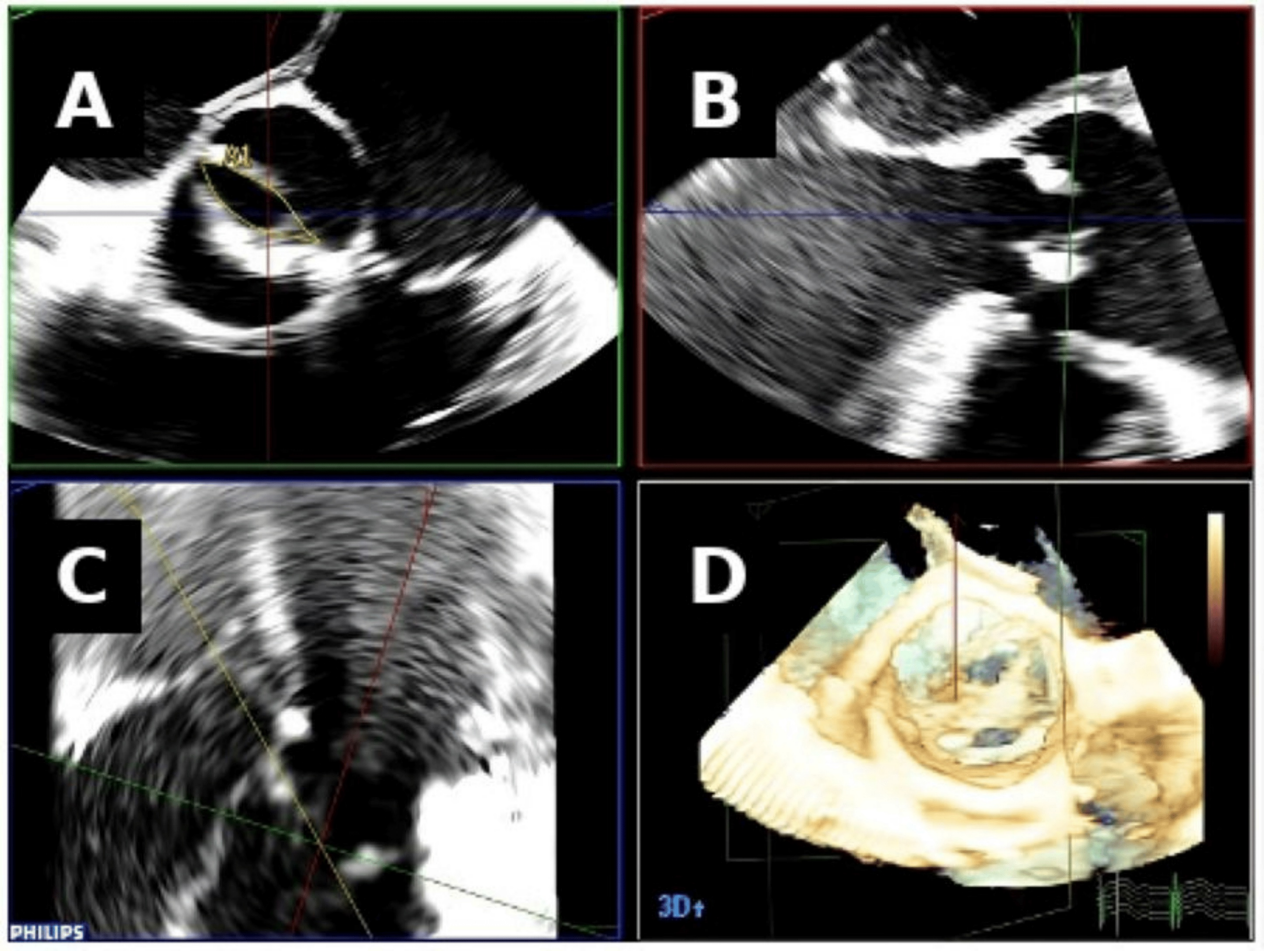
Three-dimensional transesophageal echocardiography using multiplanar reconstruction for intra-procedural assessment of bicuspid aortic valve morphology. Figure reproduced from the study by De Agustin et al. licensed under CC-BY [24].

During percutaneous aortic valve replacement, advanced imaging techniques play a key role in anatomical characterization and real-time procedural assessment. Three-dimensional transesophageal echocardiography (Figure 3) with multiplanar reconstruction allows detailed visualization of aortic valve morphology, including identification of bicuspid anatomy and accurate measurement of the valve opening area, supporting appropriate procedural planning and immediate evaluation of valve deployment.

With respect to vascular access, the transfemoral route remains the predominant approach and accounts for the vast majority of transcatheter aortic valve replacement procedures, reaching approximately 94.8% of cases in contemporary series. When femoral access is not feasible due to unfavorable vascular anatomy or severe peripheral arterial disease, alternative access routes such as the transaxillary or transcaval approaches are employed, allowing the procedure to be adapted to a broader range of patients [25]. The management of vascular access has also evolved with the widespread adoption of percutaneous closure devices. Systems such as the Perclose ProGlide™ have demonstrated safety and efficacy comparable to surgical access closure, while offering the advantages of reduced invasiveness and faster recovery [26,27].

**Figure 4:**
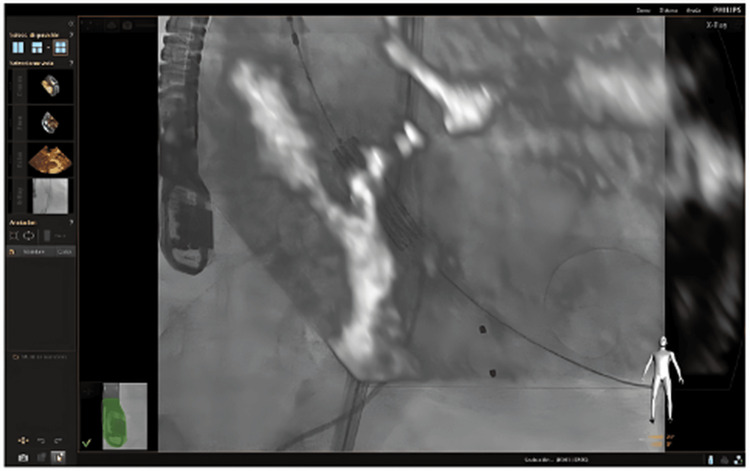
Fusion imaging-guided positioning of the transcatheter aortic valve immediately before deployment at the aortic annulus. Figure reproduced from the study by de Agustin et al. licensed under CC-BY [24].

Fusion imaging (Figure 4) provides real-time integration of imaging modalities to guide accurate transcatheter valve positioning at the aortic annulus immediately before deployment, supporting procedural precision during transcatheter aortic valve replacement.

From a hemodynamic standpoint, transcatheter aortic valve replacement leads to marked and immediate improvements in valve performance. Studies have consistently shown significant reductions in mean transaortic gradients, with values decreasing from approximately 48.7 mmHg prior to intervention to around 8.8 mmHg following valve implantation. These changes translate into favorable valve function in the vast majority of patients, with high procedural success rates and low incidence of clinically significant paravalvular regurgitation [23,28].

Despite these favorable outcomes, early complications remain an important consideration. The most frequently reported adverse events include vascular access-related complications, conduction disturbances that may necessitate permanent pacemaker implantation, and varying degrees of paravalvular leak [28,29]. However, the incidence of major vascular complications has declined substantially, with reported rates of approximately 2.3% in contemporary cohorts [23]. Other potential complications, such as stroke and bleeding, continue to be monitored closely, although their frequency has decreased over time as a result of refinements in procedural techniques, improved patient selection, and ongoing advances in device design [23,28].

Impact of percutaneous aortic valve replacement on cardiovascular prognosis

Transcatheter aortic valve replacement has been consistently associated with marked symptomatic and functional improvement in patients with severe aortic stenosis. A substantial reduction in symptom burden has been documented, with the proportion of patients classified as New York Heart Association class III or IV decreasing from 82% before the procedure to 11% after intervention. Sustained clinical benefit has also been observed over time, as approximately 77% of patients report symptomatic improvement at 12 months following transcatheter aortic valve replacement. Notably, this improvement in clinical status does not always correlate directly with changes in conventional echocardiographic parameters, suggesting that symptom relief may reflect complex functional and hemodynamic adaptations beyond standard measures. In addition, improvements in left ventricular global longitudinal strain and left atrial phasic function have been reported, indicating the presence of reverse cardiac remodeling following valve intervention [30,31].

These symptomatic gains are closely linked to significant changes in hemodynamic performance. Transcatheter aortic valve replacement results in pronounced reductions in mean transaortic valve gradients, with reported decreases from approximately 44 mmHg to 9 mmHg, reflecting effective relief of left ventricular outflow obstruction [30]. Despite these favorable hemodynamic changes, left ventricular ejection fraction does not invariably improve in all patients. However, individuals with reduced baseline left ventricular ejection fraction, particularly those with values below 40%, tend to exhibit the greatest recovery of systolic function after the procedure, underscoring the heterogeneity of ventricular response to valve replacement [31,32].

Beyond improvements in symptoms and hemodynamics, transcatheter aortic valve replacement has demonstrated a beneficial impact on clinical outcomes related to heart failure. Patients undergoing the procedure show a lower incidence of heart failure-related hospitalizations compared with those managed conservatively with medical therapy, contributing to improved overall clinical trajectories [33]. Furthermore, rapid improvement in markers of cardiac damage following transcatheter valve implantation has been associated with a reduced risk of composite endpoints that include death or hospitalization for heart failure, highlighting the prognostic relevance of early postprocedural recovery [34].

Importantly, as early mortality related to valvular obstruction declines, the determinants of long-term prognosis increasingly reflect the burden of systemic cardiovascular disease rather than residual valve dysfunction. Despite procedural success, residual cardiovascular risk persists after transcatheter aortic valve replacement. The presence of comorbid conditions such as coronary artery disease, prior myocardial infarction, myocardial fibrosis, conduction disturbances requiring permanent pacemaker implantation, and paravalvular regurgitation has been associated with attenuated ventricular recovery and adverse long-term outcomes [32,35]. These observations indicate that successful valve replacement does not eliminate the underlying substrate of cardiovascular vulnerability. Instead, improved survival following transcatheter aortic valve replacement shifts clinical focus toward structured secondary cardiovascular prevention, aimed at mitigating systemic disease progression and optimizing long-term outcomes beyond the procedural horizon.

Secondary cardiovascular prevention after percutaneous aortic valve replacement

Following successful transcatheter aortic valve replacement, long-term prognosis is increasingly determined by systemic cardiovascular disease rather than persistent valvular obstruction. As procedural mortality declines and symptomatic improvement is achieved, residual risk is primarily driven by coronary artery disease, myocardial dysfunction, arrhythmias, metabolic comorbidities, and vascular pathology. Consequently, structured secondary cardiovascular prevention becomes a central component of postprocedural care. Effective control of cardiovascular risk factors remains fundamental in this context. Management of hyperlipidemia, hypertension, and diabetes mellitus is essential to reduce residual cardiovascular risk and limit progression of systemic atherosclerotic disease. Lifestyle interventions, including regular physical activity, adherence to a cardioprotective diet, smoking cessation, and maintenance of appropriate body weight, should be systematically encouraged as part of longitudinal cardiovascular management [36]. These measures are not adjunctive but integral to optimizing outcomes in patients whose survival has been extended through valve intervention.

Optimization of pharmacological therapy is equally important, particularly in patients with coexistent heart failure or coronary artery disease. Guideline-directed medical therapy should be implemented and individualized according to patient characteristics to improve survival and reduce rehospitalization. Because transcatheter valves are bioprosthetic, antithrombotic strategies must be tailored to thromboembolic and bleeding risk rather than valve mechanics alone. Current evidence supports the preferential use of single antiplatelet therapy over dual antiplatelet therapy following transcatheter aortic valve replacement, as this approach reduces bleeding risk without increasing ischemic events. In patients requiring long-term oral anticoagulation, such as those with atrial fibrillation, simplified regimens are generally favored given the increased bleeding risk associated with combination therapy [37-39].

Beyond pharmacological management, structured follow-up facilitates early identification of atrial fibrillation, conduction disturbances, heart failure progression, and other complications that contribute to late cardiovascular events. Cardiovascular rehabilitation and sustained lifestyle modification may further support functional recovery and risk factor control in selected patients. Preventive measures also include attention to oral hygiene and regular dental surveillance to reduce the risk of infective endocarditis, which remains a serious complication after valve implantation. Taken together, secondary cardiovascular prevention after transcatheter aortic valve replacement should not be viewed as a generic extension of standard cardiovascular care, but as a necessary strategy to address the systemic disease processes that continue to shape long-term outcomes once valvular obstruction has been corrected [36].

Long-term follow-up and continuity of care

Long-term outcomes following transcatheter aortic valve replacement have been increasingly well characterized, with growing evidence supporting favorable valve durability over extended follow-up periods. Available data indicate low rates of structural valve deterioration and bioprosthetic valve failure after the procedure. In a five-year follow-up study, moderate structural valve deterioration was reported in 5.31% of patients, while severe deterioration occurred in only 0.63%, and late bioprosthetic valve failure was observed in 3.13% of cases, highlighting the overall durability of transcatheter valves in mid-term follow-up [40]. Longer-term observations in high-risk populations have demonstrated a cumulative incidence of structural valve deterioration of 9.8% at 12 years, with self-expanding valves exhibiting lower rates of deterioration compared with balloon-expandable devices, suggesting that valve design may influence long-term performance [41].

Within this context, regular echocardiographic follow-up plays an essential role in longitudinal management. Serial imaging allows early detection of changes in transvalvular gradients, valve area, and regurgitation, facilitating timely intervention when deterioration is identified. However, structured follow-up after transcatheter aortic valve replacement extends beyond prosthetic valve surveillance. As long-term survival improves, late clinical events are more frequently driven by systemic cardiovascular disease rather than structural valve failure alone. Evidence from the PARTNER 2 study demonstrating stable hemodynamic performance and sustained quality-of-life improvements over five years reinforces the concept that long-term prognosis increasingly depends on factors external to the valve itself [42].

Accordingly, continuity of care must integrate valve monitoring with comprehensive cardiovascular risk management. The OBSERVANT II trial supports a lifetime management approach in which routine follow-up is coordinated with optimization of medical therapy, detection of arrhythmias, management of heart failure progression, and control of cardiometabolic risk factors [7]. This model operationalizes secondary prevention by embedding transcatheter aortic valve replacement within a structured, multidisciplinary framework rather than treating it as a discrete procedural episode. Sustained clinical benefit also depends on consistent engagement in longitudinal care. Adherence to pharmacological therapy, scheduled follow-up, and risk factor modification contributes not only to preservation of prosthetic valve performance but also to mitigation of systemic cardiovascular risk. Long-term observational data suggest that structured follow-up programs and patient engagement are associated with improved durability of benefit and reduced late cardiovascular events [43].

## Conclusions

Degenerative aortic stenosis should be understood as a manifestation of systemic cardiovascular disease rather than an isolated valvular disorder, given its shared risk factors, molecular pathways, and pathophysiological mechanisms with atherosclerosis. Chronic inflammation, endothelial dysfunction, and osteogenic signaling contribute not only to progressive valvular degeneration but also to the high prevalence of comorbid conditions such as coronary artery disease, myocardial dysfunction, and diabetes mellitus, which substantially influence long-term prognosis. Transcatheter aortic valve replacement effectively relieves valvular obstruction and results in significant symptomatic and hemodynamic improvement, with reductions in heart failure-related hospitalizations and early mortality.

However, by correcting the dominant mechanical burden, transcatheter aortic valve replacement shifts long-term prognostic determinants toward systemic cardiovascular disease rather than eliminating residual risk. Optimal outcomes therefore depend on embedding the procedure within a structured secondary cardiovascular prevention framework that integrates meticulous preprocedural assessment, procedural optimization, individualized management of comorbidities and antithrombotic therapy, and longitudinal follow-up combining prosthetic valve surveillance with aggressive cardiovascular risk reduction. In contemporary practice, transcatheter aortic valve replacement should be viewed not as a standalone intervention, but as a pivotal structural component within an integrated, long-term cardiovascular prevention strategy.
